# Electrophysiological features and outcomes of post-infectious myoclonus-ataxia syndrome: a case report and literature review

**DOI:** 10.1186/s13256-026-06009-8

**Published:** 2026-04-20

**Authors:** Ru-Yi Wang, Yang Zheng, Yi Ge, Yong-Feng Xu, Yao Ding

**Affiliations:** 1 Department of Neurology, Second Affiliated Hospital, School of Medicine, Zhejiang University, 88 Jiefang Road, Hangzhou, 310009 China; 2 Department of Neurology, First Affiliated Hospital, Zhejiang University of Traditional Chinese Medicine, Hangzhou, China; 3 Department of Epilepsy Center, Second Affiliated Hospital, School of Medicine, Zhejiang University, 88 Jiefang Road, Hangzhou, 310009 China

**Keywords:** Myoclonus, Ataxia, COVID-19, Movement disorder, Post-infectious

## Abstract

**Background:**

Myoclonus has become one of the neurological manifestations associated with coronavirus disease 2019 (COVID-19); however, the origin and pathophysiological mechanism remained uncertain.

**Case presentation:**

A rare case of myoclonus-ataxia syndrome associated with COVID-19 was presented**.** A 52-year-old Asian woman exhibited generalized myoclonus, ataxia, nystagmus, and dysarthria two weeks after a fever episode, which deteriorated rapidly within a few days. Electromyography (EMG) revealed synchronized bursts in both hands concurrent with myoclonic jerks. The absence of somatosensory evoked potential and lack of correlation between electromyography (EEG) and EMG suggested a subcortical origin of the myoclonus. A post-infectious immune-mediated process was considered the most likely mechanism, given the latency from fever to myoclonus onset, and the absence of other possible etiologies. Treatment with intravenous methylprednisolone led to notable improvement and a good outcome at the two-month follow-up.

**Conclusion:**

Myoclonus arising from subcortical structures can be associated with COVID-19. Early aggressive immunotherapy is important for a favorable outcome. Review of similar cases along with our report suggests COVID-19-associated myoclonus-ataxia as a distinct syndrome warranting prompt diagnosis and treatment.

**Supplementary Information:**

The online version contains supplementary material available at 10.1186/s13256-026-06009-8.

## Background

With the wide spread of the severe acute respiratory syndrome coronavirus 2 (SARS-CoV-2), the spectrum of neurological manifestations associated with COVID-19 has significantly expanded [1]. The commonly reported neurological disorders include intracerebral hemorrhages, encephalitis, acute myelitis, anosmia/ageusia, Guillain–Barré syndrome [2]. Among the diverse symptoms, myoclonus has garnered relatively little attention and has been infrequently reported, particularly when in conjunction with ataxia. Previous reports described myoclonus associated with COVID-19 are diverse, and manifest as spontaneous or action induced, multifocal or generalized, positive or negative myoclonus, affecting axial or proximal limb muscles [3], and the severity of myoclonus ranges from mild cases that can be managed on an outpatient basis [4] to severe cases that necessitate hospitalization. Furthermore, the origin and pathophysiological mechanism of myoclonus remain undetermined in the absence of adequate evidence, particularly EEG and EMG. Herein, we present a case of myoclonus status accompanied by ataxia after COVID-19 infection. The mechanisms leading to the syndrome were analyzed. We also summarized previous reports of COVID-19-associated myoclonus, delineating the clinical pearls warranting prompt diagnosis and treatment.

## Case presentation

A 52-year-old Asian woman visited the local clinic due to a two-day history of low-grade fever, widespread body pain, fatigue, occasional daytime cough, and mild diarrhea. She was subsequently diagnosed with COVID-19 and received symptomatic treatment including Ibuprofen and Dextromethorphan. Approximately two weeks after her initial symptoms, the patient noticed spontaneous jerking movements in her proximal extremities and trunk. These jerks were exacerbated during voluntary actions and were sensitive to tactile and auditory stimuli, leading to an unsteady gait. Over the next few days, the jerking gradually escalated, peaking in severity on the fifth day from the onset, accompanied by loss of the ability to walk and slurred speech.

Concerned about her deteriorating ability to walk, the patient visited the emergency department of our hospital. The patient reported no symptoms of nausea, vomiting, vertigo, dyspnea, changes in taste or smell, or cognitive impairment. Neurological examinations revealed myoclonus primarily affecting the distal upper limbs, along with wide-based gait and ataxia. The patient exhibited impaired rapid alternating hand movements and dysmetria in the upper limbs during finger-to-nose tests, as well as dysarthria (*see *Additional file 1). Additionally, coarse horizontal nystagmus was observed. Sensations, deep tendon reflexes, and plantar responses appeared normal. Other findings were unremarkable during the physical examination.

**Fig. 1:**
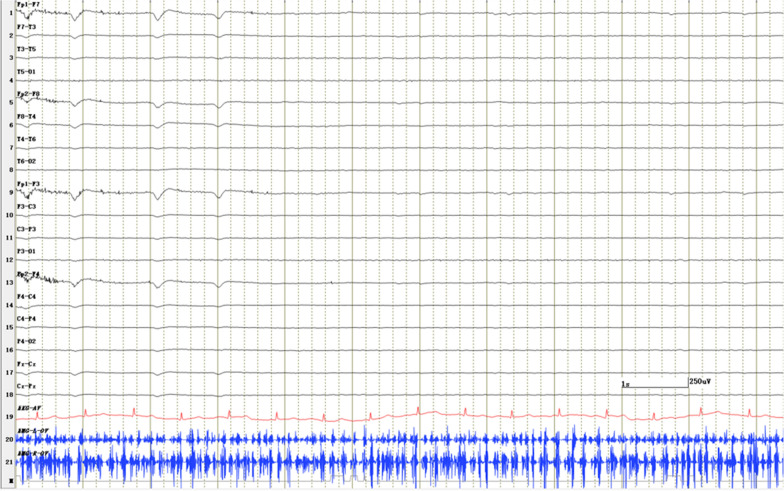
EMG showing rhythmic bursts in both hands that coincided with the myoclonic jerks. Video-EEG monitoring showed no abnormalities occurring simultaneously with myoclonic jerks

EMG displayed rhythmic bursts in both hands, occurring simultaneously and synchronized with the myoclonic jerks, while EEG revealed no significant abnormalities (Fig. 1). In addition, the absence of jerk-locked cortical potential on back averaging provided additional evidence of the subcortical origin of the myoclonus. Neither magnetic resonance imaging (MRI) nor positron emission tomography-computed tomography (PET-CT) conducted after the onset yielded remarkable results.

Chest computed tomography (CT) displayed no notable abnormalities. Cerebrospinal fluid (CSF) analysis, including biochemistry, cytology, and autoimmune encephalitis antibodies, yielded negative results. Demyelinating markers (including aquaporin-4 antibody, myelin oligodendrocyte glycoprotein antibody, and glial fibrillary acidic protein) and paraneoplastic antibody were undetectable in blood samples. Other serum markers, including leukocyte count, ferritin, lactate dehydrogenase, and C-reactive protein, all fell within the normal ranges. No other evidence of infection or autoimmune diseases was found.

A post-infectious immune-mediated process was considered the most likely mechanism, given the latency from fever to myoclonus onset, and the absence of other possible etiologies. The patient declined intravenous immunoglobulin (IVIG) treatment due to financial constraints. She received high-dose corticosteroid therapy with intravenous methylprednisolone (1 g/day), which was gradually tapered to 40 mg/day at discharge, to be tapered off within one month. Clonazepam and levetiracetam were also introduced to manage the myoclonus, leading to gradual and consistent improvement in her clinical condition. The patient was discharged three weeks after admission with significant improvement in movement and coordination. At the two-month follow-up, she reported a remarkable 90% alleviation of symptoms and remained medication free.

## Discussion

Around 10%–30% of COVID-19 patients experience neurological symptoms during the acute phase. Strikingly, neurological dysfunctions may serve as the initial sign of infection, sometimes even without respiratory-related symptoms [5]. Myoclonus has been increasingly recognized as a neurological presentation associated with COVID-19 and one of the post-infectious immune-mediated complications [3–9]. Myoclonus can be categorized based on its anatomical substrates, which include peripheral, spinal (segmental and propriospinal), subcortical, and cortical forms [10]. In the present case, the myoclonus was generalized, positive, and sensitive to stimuli. Notably, neurophysiological assessments revealed the absence of both giant somatosensory evoked potential and an EEG-EMG correlation. These findings suggest that the myoclonus may arise from subcortical structures, with predominant involvement of the brainstem. The presence of ataxia and ocular flutter during eye movement evaluation also indicated cerebellar involvement. Thus, the concurrent presence of myoclonus with ataxia constitutes a distinct myoclonus-ataxia syndrome in which these cooccurring symptoms are synchronized. These cases account for a unique subset within the spectrum of neurological manifestations associated with COVID-19. We provide a list of case reports featuring likely myoclonus-ataxia syndrome associated with COVID-19 in Table 1.

**Table 1:** Clinical features, ancillary findings, and prognosis of COVID-19-associated myoclonus-ataxia syndrome

**Study**	**Age (years), sex**	Latency*	**Neurological features**	**Myoclonus origin**	**CSF**	**Serum**	**Neuroimage**	**Treatment and response**	**Follow-up**
Muccioli et al [3]	58, M	21 days	•Multifocal myoclonus, predominant in the right proximal inferior limb muscles (sensitive to tactile and action stimuli) •Markedly agitated (before myoclonus)	Subcortical, possibly secondary to brainstem involvement	Elevated leukocytes and protein levels, slightly elevated CSF IL-6 level	An elevated IL-8 CSF/blood	MRI showed cerebral small-vessel disease of moderate severity	LEV, CLN (marked amelioration of myoclonus within 5 days)	28 days (marked amelioration of the myoclonus)
Schellekens et al [4]	48, M	13 days	•Generalized myoclonic jerks particularly involving the hands (not sensitive to tactile and auditory stimuli) •Cerebellar ataxia of the arms and legs and an ataxic gait; saccadic intrusions and hypermetric saccades	Uncertain	N	N	N	LEV (alleviated the myoclonus within several days.)	62 days (both the myoclonus and the ataxia had improved, but recovery was not yet complete)
Dina Ben Mohamed et al [5]	60, M	following the COVID vaccination	•Multifocal myoclonic jerks (sensitive to tactile and auditory stimuli) •Abnormal gait without cerebellar ataxia, mild dysarthria •Paralysis of Down-Gaze	Uncertain	N	N	N	Dexamethasone for 3 days, along with CLN and LEV	3 days (started improving)
Shetty et al [6]	41, M	10 days	•Generalized severe myoclonus, predominantly proximal (sensitive to tactile and auditory stimuli) •Truncal ataxia; gait ataxia •Mild frontal dysfunction	Subcortical	N	N	N	LEV, CLN (mild improvement); IVMP 1 g/day given for 5 days (significant improvement);	6 weeks (complete resolution of ataxia and near total resolution of myoclonus)
Asan et al [8]	44, M	14 days	•Severe generalized action myoclonus affecting his speech •A impairment of speech, an unsteady gait and limb clumsiness •Dysgraphia	Uncertain	N	GFAP-serum antibodies titer of 1:1000	N	IVMP1 g/d for 5 d(improved)	3 months (showed no residual neurological symptoms)
Dijkstra et al [9]	44, M	14 days	•Multifocal action-induced myoclonic jerks (sensitive to tactile and auditory stimuli) •Prominent gait ataxia and titubation, slurred speech horizontal saccadic intrusions and a transient ocular flutter, no clear opsoclonus •Attention and memory deficits, perseveration, impulsivity, some anxiety, hypervigilance, and insomnia	Uncertain	N	N	N	IVMP1 g/d for 5 d(slow recuperation); IVIG 0.4 g/kg for 3 days	2 months (full recovery)
Grimaldi et al [12]	72, M	17 days	•Spontaneous diffuse myoclonus (stimulus-sensitive) •Ataxia, and upper limb dysmetria, dysarthria	Uncertain	Absence of CSF-specific IgG oligoclonal banding	High titers of IgG autoantibodies (1/25,000,1/96 in CSF)	PET showed diffuse cortical hypometabolism associated with putaminal and cerebellum hypermetabolism	IVIG 0.4 g/kg/d for 5d (no improvement); IVMP1 g/d for 5 d(improved); CLN0.3 mg tid (stopped for drowsiness)	37 days (discharged from the hospital)

*LEV* levetiracetam, *CLN* clonazepam, *M* male, *F* female, *IVMP* intravenous methylprednisolone, *IVIG* intravenous immunoglobulins, *N* normal

Latency*: from COVID-19-related systemic symptoms onset to neurological symptoms onset

Several hypotheses have been proposed to explain the underlying mechanisms of COVID-19-associated myoclonus. These include the following: (1) anoxic brain injury due to respiratory restriction or stroke; (2) direct virus invasion of the central nervous system; (3) cytokine-triggered neuroinflammation; and (4) adverse drug reactions, such as those provoked by medications like propofol or opioids (e.g., fentanyl), or serotonin syndrome induced by lopinavir/ritonavir [3]. In the current case, there was no evidence of hypoxia or intracranial infection, and the patient had no history of specific drug usage. Given the approximate two-week latency between fever onset and the appearance of neurological symptoms, cytokine-mediated neuroinflammation emerges as the primary consideration in our case. Although no tissue test was performed in our case, a recent study associated with COVID-19 demonstrated CSF antineuronal immunoglobulin (Ig)Gs in mouse brain tissue through indirect immunohistochemistry(IHC), especially astrocytic staining pattern was only observed in the patients with myoclonus, indicating autoimmune involvement in the pathophysiological mechanism of post-COVID-19 myoclonus [11].

The treatment strategies for myoclonus and ataxia associated with COVID-19 primarily focus on symptomatic management and immunotherapy. Symptomatic management includes the use of anti-epileptic medications such as clonazepam or levetiracetam, which have been effective in alleviating myoclonus [13]. Immunomodulatory treatment is also recommended, especially for post-infectious or para-infectious cases, to accelerate recovery. This includes corticosteroids (e.g., IVMP), IVIG, or plasma exchange. [6, 13, 14] The patient finally experienced significant improvement (over 90%) with the administration of IVMP and clonazepam. It is also worth noting that all cases reported in Table 1 showed substantial improvement with immunomodulatory treatment. However, the pace of recovery and drug responsiveness varied among patients, with some individuals responding favorably to anti-myoclonic medication alone, while others required an escalation of treatment to include plasma exchange due to a lack of improvement from anti-myoclonic and steroid medication. More studies are needed to standardize the diagnosis and treatment of the myoclonus-ataxia syndrome.

## Conclusion

The myoclonus-ataxia syndrome has been recognized as a distinct post-infectious immune-mediated complication associated with COVID-19, and it appears to be infrequent in other viral infections. Neurophysiological assessments in our case suggest that the myoclonus originates from subcortical structures. Post-infectious autoimmune involvement was suggested, emphasizing the importance of early aggressive immunotherapy for a favorable outcome.

## Supplementary Information

Additional file 1: Neurological examinations in the acute phase of the illness revealed myoclonus primarily affecting the distal upper limbs, along with wide-based gait and ataxia. The patient also exhibited slurred speech as well as impaired rapid alternating hand movements.

## Data Availability

Please contact the corresponding author for data requests.

## Abbreviations

COVID-19: Coronavirus disease 2019
EMG: Electromyography
EEG: Electromyography
SARS-CoV-2: Severe acute respiratory syndrome respiratory coronavirus 2
MRI: Magnetic resonance imaging
PET-CT: Positron emission tomography-computed tomography
CT: Computed tomography
CSF: Cerebrospinal fluid
IVIG: Intravenous immunoglobulin
IVMP: Intravenous methylprednisolone
IHC: Indirect immunohistochemistry
